# Assessment of ESGO Quality Indicators and Factors Associated with Recurrence Following Surgery for Early-Stage Cervical Cancer: A Retrospective Cohort Study

**DOI:** 10.3390/jcm14197041

**Published:** 2025-10-05

**Authors:** María Espías-Alonso, Mikel Gorostidi, Ignacio Zapardiel, Myriam Gracia

**Affiliations:** 1 Gynecologic Oncology Unit, La Paz University Hospital, Paseo de la Castellana 261, 28046 Madrid, Spain; mariaalonsoespias@gmail.com (M.E.-A.); ignaciozapardiel@hotmail.com (I.Z.); dra_gracia@hotmail.com (M.G.); 2 Gynecologic Oncology Unit, Donostia University Hospital, Begiristain Doktorea Pasealekua S/N, 20014 Donostia, Spain; 3 Biogipuzkoa Health Research Institute, 20014 Donostia, Spain; 4 Faculty of Medicine, Basque Country University, 48940 Leioa, Spain

**Keywords:** cervical cancer, quality of treatment, survival, oncological outcome

## Abstract

**Background/Objectives**: In 2019, the European Society of Gynaecological Oncology (ESGO) published a set of quality indicators (QIs) for the surgical management of cervical cancer with the aim of improving clinical practice. The objective of this study is to evaluate the influence of ESGO QIs and clinicopathological factors on progression-free survival (PFS) in patients with early-stage cervical cancer in a retrospective cohort. **Methods**: A retrospective study was conducted in patients with early-stage cervical cancer who underwent radical surgery with pelvic lymph node assessment at La Paz University Hospital between 2005 and 2022. The cohort was divided into two groups according to the timing of surgery (before vs. after 2010), when MRI was implemented as a standardized diagnostic tool and the multidisciplinary tumor board was established. Univariate and multivariate Cox regression analyses were performed, including demographic and histopathological variables, as well as adherence to ESGO QIs, focusing on those related to the overall management. Hazard ratios and 95% confidence intervals were estimated. Kaplan–Meier survival curves were generated and compared between groups. **Results**: The implementation of systematic MRI and a multidisciplinary tumor board at our center was associated with a significant reduction in positive surgical margins (*p* = 0.003) and parametrial invasion (*p* < 0.001), as well as improved diagnostic accuracy, lowering the rate of upstaging from 31.6% before 2010 to 4.4% thereafter (*p* < 0.001). PFS in the post-2010 cohort was significantly improved (log-rank *p* = 0.0408), although no differences in overall survival (OS) were observed (log-rank *p* = 0.2602). Additionally, cervical conization prior to radical hysterectomy was associated with a markedly reduced risk of recurrence (HR 0.12, *p* < 0.001), representing the most significant prognostic factor for PFS in our cohort. **Conclusions**: The correct application of ESGO QIs, along with appropriate staging and pathological assessment, is essential to improve prognosis in cervical cancer. Systematic implementation of these standards is recommended to optimize clinical care.

## 1. Introduction

Cervical cancer ranks as the fourth most common malignancy and the fourth leading cause of cancer-related mortality among women globally [1]. Five-year relative survival varies among European countries, ranging from 57% in Eastern Europe to 67% in Northern Europe [2]. This observation also highlights disparities in screening availability and human papillomavirus (HPV) prevalence, as well as variations in surgical care quality, diagnostic approaches, and the administration of adjuvant treatments [3].

In early-stages, surgery remains the treatment of choice, with radical hysterectomy combined with pelvic lymph node staging established as the standard procedure [4]. However, according to the most recent update of the European Society of Gynaecological Oncology (ESGO) guidelines, less radical procedures such as cervical conization or simple hysterectomy may be considered appropriate alternatives in very early stages, particularly in carefully selected patients [4]. Regarding nodal assessment, bilateral pelvic lymphadenectomy (PL) continues to be regarded as the reference standard. However, sentinel lymph node biopsy (SLNB) is strongly recommended, and in certain cases may replace systematic PL as the sole method of nodal staging, thereby reducing morbidity without compromising oncological safety [4].

Improved surgical quality has been associated with better outcomes in patients with other malignancies, including breast, lung, gastric, colorectal, soft tissue sarcoma, and ovarian cancers. In this type of tumor patients receiving not adherent care to European Society of Medical Oncology guidelines experienced a risk of dead more than 100% compared with patients treated with optimal care [5,6].

In an effort to standardize surgical practice and improve oncologic outcomes, ESGO introduced in 2019 a comprehensive set of fifteen quality indicators (QIs) [3]. These indicators cover structural, process, and outcome domains, and their systematic implementation is expected to enhance the quality and consistency of surgical management in cervical cancer across institutions, improving the level of care, while ensuring equity and safety in treatment. Among these indicators, multidisciplinary case evaluation within a tumor board and the systematic use of preoperative magnetic resonance imaging (MRI) have been shown to be key factors for appropriate therapeutic planning and prognosis.

Although some studies have evaluated the outcomes of ESGO QIs after radical hysterectomy in retrospective cohorts, data on the impact of their implementation is still lacking [7,8]. Furthermore, data from single-center cohorts can provide valuable insight into real-world implementation of these guidelines, highlight areas for improvement, and support quality assurance initiatives.

The objective of this study was to evaluate the impact of ESGO QIs and clinicopathological factors on progression-free survival (PFS) in patients with early-stage cervical cancer within a retrospective cohort.

## 2. Materials and Methods

### 2.1. Study Design and Data Selection

This retrospective observational study included 128 patients diagnosed with early-stage cervical carcinoma (2018 International Federation of Gynecology and Obstetrics [FIGO] stages IA1 with lymph vascular space invasion [ILV] to IB2 or IIA1) [9], who underwent radical surgery (hysterectomy, trachelectomy, or parametrectomy) with pelvic lymph node assessment at La Paz University Hospital between January 2005 and December 2022. In our cohort, nodal staging was performed using PL, SLNB, or both, depending on the time period. Before 2011, SLNB had not yet been introduced in our center, and exclusive PL was the standard of care. Between 2011 and 2016, the SLNB technique was implemented, and SLNB combined with systematic PL was performed. Since 2017, as our center has been participating in the SENTIX trial [10], exclusive SLNB has been used for lymph node staging.

All procedures were performed or supervised by gynecologic oncologists, and all histological subtypes were eligible. Exclusion criteria included: patients with suspicious lymph nodes on preoperative imaging (enlarged, firm, or necrotic), advanced disease stages, abandonment of the surgical procedure, non-radical interventions, or incomplete clinical records. Before the publication of the LACC trial in 2018 [11], the use of uterine manipulators and minimally invasive surgery as the main surgical approach was common practice. From that point onward, however, uterine manipulators were no longer used, and patients with tumors larger than 2 cm were treated with open surgery. Adjuvant treatment with external beam radiation (EBRT), chemotherapy (CT), and/or brachytherapy (BT) was administered selectively, according to the institutional tumor board’s recommendations, based on histopathologic risk factors and individual patient characteristics. Pelvic irradiation was delivered at a total dose of 45–50.4 Gy. When chemotherapy was indicated, weekly cisplatin (40 mg/m^2^) was employed. Data collection was carried out in an Excel spreadsheet through retrospective review of medical records after approval by the Ethics Committee of La Paz University Hospital (reference PI-3668), including tumor characteristics, preoperative assessment, type of surgery performed, histopathological data, adjuvant treatment administered, and follow-up.

Data on the ESGO QIs met by each patient were recorded. Among the fifteen QIs, only six could be assessed at the individual level, as the remaining indicators referred to proportions of patients treated at the center or within a specific time period. For these proportional indicators, data were also collected, and one point was assigned to each patient if the indicator was met in the corresponding yearly block.

From 2010 our center systematically implemented several guideline-based practices currently considered good clinical practice in cervical cancer, including standardized pelvic MRI for initial staging, multidisciplinary tumor board discussions, and active participation in clinical trials. Therefore, 2010 was chosen as the cutoff to assess survival differences, with the cohort divided into two groups: surgery before 2010 and surgery in 2010 or later.

PFS was defined as the time from the end of treatment to the diagnosis of recurrence (local or metastatic). Overall survival (OS) was defined as the time from the end of treatment to the date of death.

### 2.2. Statistical Analysis

Descriptive statistics were performed. Continuous variables were summarized as mean ± standard deviation (SD) or as median and interquartile range (IQR) when distributions were non-parametric; comparisons were made using Student’s *t*-test or the Mann–Whitney U test, respectively. Categorical variables were described as frequencies and percentages, and compared using Pearson’s chi-square or Fisher’s exact test. PFS and OS were estimated with the Kaplan–Meier method, and survival curves were compared with the log-rank test. Logistic regression was used for univariate analysis to identify potential predictors of recurrence, including demographic, histologic and adherence to ESGO criteria variables. Variables with *p* ≤ 0.30 were entered into a multivariate logistic regression model with backward stepwise selection. The univariate comparisons were considered exploratory; variables with *p* ≤ 0.30 were entered into the Cox multivariable model (with PFS as the primary endpoint). No strict multiplicity corrections were applied, in order to avoid inflating type II error in this limited cohort; statistical significance was set at *p* < 0.05. Data were entered into Excel and all analyses were conducted using STATA 15.0 (StataCorp LP, College Station, TX, USA).

## 3. Results

**Figure 1:**
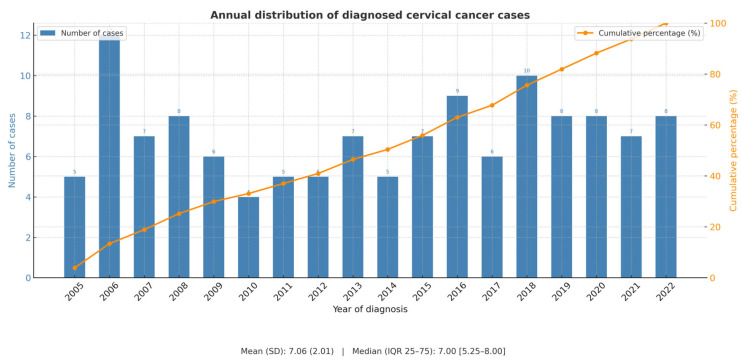
Annual distribution of patients undergoing surgery for early-stage cervical cancer at our center.

Of our initial cohort of 134 patients, a total of 128 (95.5%) eligible patients were included in the study, with a median number of patients undergoing surgery per year at our center of 7.1 (SD 2.1) (Figure 1). The median age in the cohort was 47.5 years (SD 11.6) and the most frequent histological subtype was squamous cell carcinoma in 81 (63.3%) patients. Pelvic MRI was used as the sole initial staging method in 43 (33.6%) patients, combined with high-resolution ultrasound in 31 (24.2%) patients, with computed tomography (CT) scan in 12 (9.4%) patients, or with both CT scan and ultrasound in 12 (9.4%) patients. In 68 (53.1%) cases, the FIGO stage at diagnosis was IB2, followed by IB1 in 46 (35.9%) cases. All the surgeries were performed or supervised by a gynecologic oncologist. Radical hysterectomy was carried out in 114 (89.1%) patients, compared to 12 (9.4%) radical trachelectomies and 2 (1.6%) radical parametrectomies, and 66 (51.6%) patients had a previous conization. Minimally invasive surgery was the most frequently used approach in 107 cases (83.6%). At the final pathological examination, 6 (4.7%) patients had parametrial invasion, 6 (4.7%) had positive margins, and 13 (10.2%) had positive lymph nodes. The histological analysis revealed that 29 (22.7%) patients had a higher stage than preoperatively diagnosed: 13 (10.2%) with stage IB3, 3 (2.3%) with stage IIB, and 13 (10.2%) with stage IIIC1. Regarding postoperative complications, the fistula rate was 2.4% (data available for 126 of the 128 patients in the cohort). Forty-five (35.5%) patients received adjuvant treatment: 23 (18%) external beam radiation (EBR) ± vaginal brachytherapy (BT), 18 (14%) concurrent chemoradiation therapy and 4 (3.1%) patients received exclusive vaginal BT. The median follow-up time was 79.7 ± 53.5 months, with a recurrence rate of 21.1%.

### 3.1. Evaluation of ESGO QIs at Our Center

**QIs related to caseload in the center, and training and experience of the surgeon:**

Regarding the QIs associated with caseload in the center and training and experience of the surgeon, our center did not reach QI 1 “*Number of radical procedures in cervical cancer performed per center per year*” (minimum target ≥ 15), although every procedure was performed or supervised by a gynecologic oncologist, so QI 2 “*Surgery performed or supervised by a certified gynecologic oncologist or a trained surgeon dedicated to gynecological cancer*” was met (target 100%).

**QIs related to the overall management:**

At our center, QI 3 “*Center participating in ongoing clinical trials in gynecological cancer*” (target ≥ 1) has been met since 2010; however, it was not possible to evaluate data prior to this year, as no adequate records of this parameter were available before then. As mentioned before, QI 4 “*Treatment discussed at a multidisciplinary team meeting*” (target 100%) has been systematically implemented at our center since 2010, when a multidisciplinary gynecologic oncology tumor board was established, composed of gynecologic oncologists, medical oncologists, radiation oncologists, radiologists, nuclear medicine specialists, and pathologists. From that year onward, weekly meetings were held, and all cases were discussed both preoperatively and postoperatively.

According to ESGO-ESTRO-ESP guidelines, preoperative work-up in early-stage cervical cancer requires the systematic use of pelvic MRI. For this reason, QI 5 “*Required pre-operative investigation*” (target 100%) has been fulfilled since 2010, but not before, as it was not standardized and was not used in 100% of cases prior to that year. The overall compliance rate for this QI in our cohort was 79%.

**QIs related to recording patient information:**

QI 6 “*Minimum required elements in surgical reports*” (target 100%) was not achieved in our cohort, as the overall compliance rate was 57%.

Regarding QI 7 “*Minimum required elements in pathology and pathology reports*” (target ≥ 90%), although most reports include 12 of the 13 essential required parameters (macroscopic description of the specimen and the tumor, tumor measurements, tumor histology and grade, presence/absence of LVSI, status of margins and lymph nodes, etc.), one parameter (length of parametrial tissue in two dimensions) is never reported, and therefore this QI was considered never met.

QI 8 “*Structured prospective reporting of the follow-up and 30-day post-operative morbidity*” (target ≥ 90%), was not met because structured prospective reporting of follow-up and 30-day postoperative morbidity was not performed at our center. However, during each follow-up visit, patients were systematically questioned about potential adverse effects, which were documented in the medical record. This QI was considered unmet in all cases.

**QIs related to the quality of surgical procedures:**

The fistula rate in our cohort was 2.4%, indicating that QI 9 “*Urological fistula rate within 30-post-operative days after a radical parametrectomy*” (target ≤ 3%) was met.

The “*Proportion of patients after primary surgical treatment who have clear vaginal (invasive disease) and parametrial margins*”, corresponding to QI 10 (target ≥ 97%), was 95.3%, and the “*Proportion of patients with a stage T1b disease T-upstaged after surgery*” (QI 11, target < 10%) was 32.8%, thus, neither of these two QI was met in our cohort. However, when analyzing the subgroup of patients operated in 2010 or later, the QI was achieved, as the positive margin rate in this group was 1.1%, with 0% parametrial invasion.

QI 12 refers to “*Recurrence rate at 2 years in patients with a stage pT1b1 with negative lymph nodes after primary surgical treatment*” (target < 10%), with a rate of 9.97% in our cohort, just below the established threshold.

**QIs related to the compliance of management with the standards of care:**

QI 13 corresponds to “*Proportion of patients with a stage T1 disease treated with primary surgery who have undergone lymph node staging according to the ESGO-ESTRO-ESP guidelines*” (target ≥ 98%). In our study, 100% of patients underwent adequate lymph node staging.

QI 14 “*Counseling about a possibility of FST*” *(target 100%)* was offered in cases meeting the criteria established by international clinical guidelines, as evidenced by the inclusion of 12 radical trachelectomies in the cohort.

Finally, according to QI 15 “*Proportion of patients receiving adjuvant chemoradiotherapy after a primary surgical treatment for a stage pT1b1pN0 disease*” the target should be less than 15%. In our cohort, this proportion was 3.13%, considerably below the recommended threshold, indicating appropriate adherence to treatment guidelines.

Table 1 summarizes the compliance of our center with the 15 ESGO QIs for cervical cancer surgery, including the established target for each indicator and the corresponding results from our cohort.

### 3.2. Comparative Analysis by Study Groups (Before 2010 vs. 2010 and Beyond)

As previously mentioned in Section 2, we stablished 2010 as cutoff for comparing differences, since three of the QIs (pelvic MRI as preoperative investigating tool, multidisciplinary tumor board, and participation in clinical trials) were implemented at our center from this time onward. Table 2 summarizes the baseline, surgical, and pathological characteristics of the different groups according to this temporal cutoff. Statistically significant differences were observed between groups in the type of parametrectomy (type C1 was more frequent before 2010, *p* < 0.001), the maximum size per image (greater in the pre-2010 group, *p* = 0.0495), previous conization (more frequent in the pre-2010 group, 63.2% vs. 42.2%, *p* = 0.035), and the presence of LVSI (higher in the pre-2010 group). No differences were found in other histological or clinical parameters.

Regarding complications, no differences were found in the rate of complications beyond 30 days (*p* = 0.575). However, intraoperative complications were more frequent in the pre-2010 group (15.8%) compared with the post-2010 group (5.6%, *p* = 0.047), reaching significant differences. In addition, the severity of complications was significantly higher before 2010 (*p* = 0.003).

**Figure 2:**
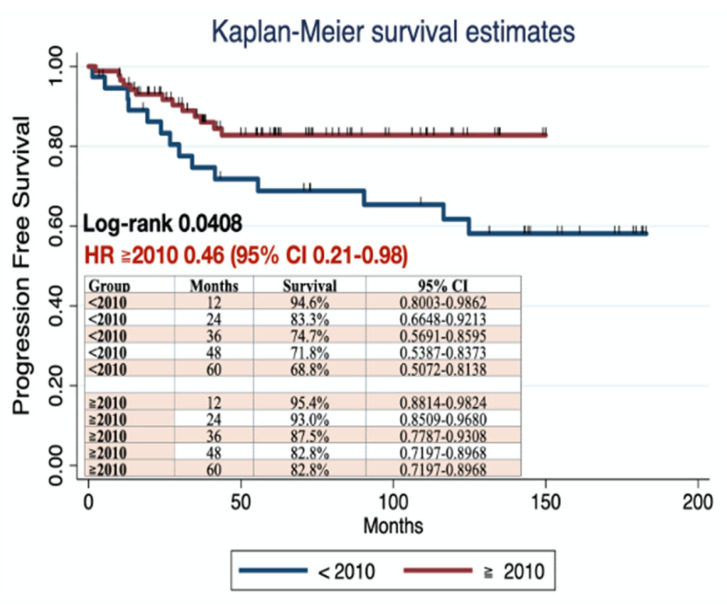
PFS comparing patients who underwent surgery before and in 2010 or after. (STATA Statistics/Data Analysis (StataCorp LP, College Station TX, USA) was used to create the figures.)

**Figure 3:**
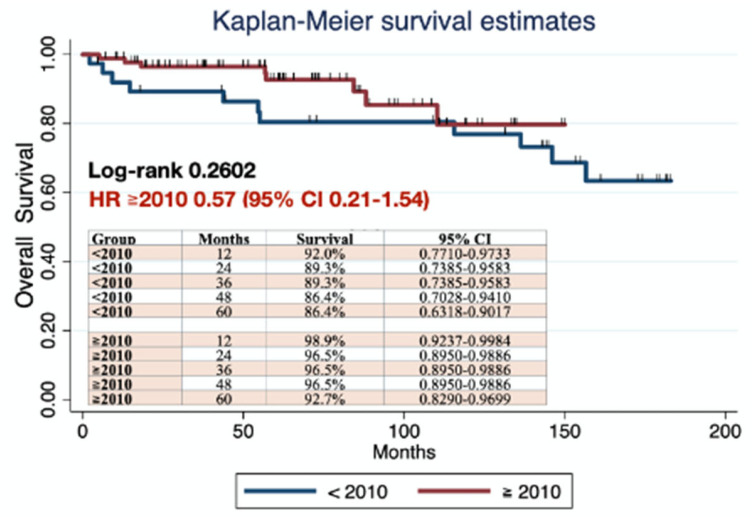
OS comparing patients who underwent surgery before and in 2010 or after. (STATA Statistics/Data Analysis (StataCorp LP, College Station TX, USA) was used to create the figures.)

In the survival analysis, statistically significant differences were found in PFS between patients who underwent surgery before and after 2010 (log-rank 0.0408) (Figure 2), with a HR for the group post-2010 of 0.46 (95% IC 0.21–0.98). No statistically significant differences were found in OS between groups (log-rank 0.2602; HR 0.55, 95% IC 0.21–1.54) (Figure 3).

Regarding parametrial invasion, significant differences were observed in the presence of parametrial involvement in the surgical specimen after radical hysterectomy: no cases were reported after 2010, whereas 6 cases (15.8%) were found before 2010 (*p* < 0.001). Similarly, significant differences were also observed in margin involvement, with only one case (1.1%) after 2010 compared to 5 cases (13.2%) before 2010 (*p* = 0.003). Moreover, analyzing patients who met the high-risk criteria for recurrence [12] significant differences were also observed, with a higher proportion in the pre-2010 group (31.3% vs. 9.4%, *p* = 0.003). Postoperative upstaging was analyzed. Upstaging occurred in 31.6% of patients in the pre-2010 group compared to 4.4% in the 2010 or later group, a difference that was statistically significant (*p* < 0.001).

We also analyzed whether adjuvant treatment was administered in patients meeting Peters’ criteria [12]. Treatment was considered adequate when patients with any high-risk criteria received postoperative chemoradiotherapy (CRT), and inadequate otherwise. No differences were found between groups (7.9% before 2010 vs. 6.8% ≥ 2010, *p* = 0.804).

### 3.3. Analysis of Factors Associated with Recurrence in Patients Who Underwent Radical Surgery for Early-Stage Cervical Cancer

To identify variables associated with a higher recurrence rate, a logistic regression analysis was conducted (Table 3). This analysis revealed that the presence of LVSI (*p* = 0.001), positive surgical margins (*p* < 0.001), parametrial involvement (*p* = 0.004), and deep stromal invasion (*p* = 0.009) were associated with an increased risk of recurrence. In contrast, cervical conization prior to radical hysterectomy emerged as a protective factor against recurrence (*p* = 0.001). In the multivariate analysis, however, only cervical conization remained statistically significant as a protective factor for recurrence, with an HR of 0.12 (95% CI 0.02–0.73, *p* = 0.021).

## 4. Discussion

This retrospective single-center study evaluates adherence to ESGO QIs and clinicopathological factors influencing PFS in patients with early-stage cervical cancer who underwent radical surgery over a 17-year period in a third-level hospital. Our findings highlight the prognostic importance of pathological features, particularly postoperative FIGO stage, LVSI, and margin status, and suggest that adherence to selected QIs, including multidisciplinary tumor board review and systematic preoperative MRI, may contribute to improved outcomes. Moreover, we found that cervical conization previous to radical hysterectomy is associated with a reduced risk of recurrence.

ESGO introduced its QIs in 2019 with the goal of standardizing surgical practice, ensuring consistent reporting, and improving outcomes across Europe [3]. While some of these indicators, such as surgical volume per center (QI 1), remain challenging to achieve in many institutions, adherence to others—such as QI 2 (specialist surgeon involvement), QI 4 (multidisciplinary tumor board discussion), and QI 5 (preoperative imaging)—is feasible and directly impacts staging accuracy, surgical planning, and decision-making.

It is well established that hospital surgical volume significantly impacts perioperative outcomes. High-volume cervical cancer centers are considered a favorable prognostic factor, being associated with improved perioperative results and lower complication rates [13,14,15]. Furthermore, the risk of recurrence is reduced when procedures are performed by experienced gynecologic oncologic surgeons [16,17]. Although our center achieves adequate surgical volumes for other gynecologic malignancies, such as endometrial or ovarian cancer [5,18], the low prevalence of cervical cancer in our region prevents us from reaching the minimum number of radical procedures recommended. Nevertheless, all radical hysterectomies are consistently performed or supervised by subspecialists in gynecologic oncology. Moreover, our result of 100% surgeries performed by gynecologic oncologists resonates with other national studies which reported 100% compliance for this indicator and similar adherence to surgical and pathology reporting standards [19].

Among the ESGO quality indicators, those related to overall management (QIs 3–5), were systematically implemented at our center in 2010. For this reason, these indicators were examined in greater detail in our study, using comparative groups to assess their impact. Our analysis identified notable improvements in surgical and pathological outcomes after the implementation of standardized MRI as preoperative staging and regular tumor-board discussions in 2010, reflected by a significant reduction in parametrial involvement, margin positivity, and postoperative upstaging rates. Accurate identification of patients eligible for surgery is particularly important in cervical cancer, as locally advanced stages do not benefit from surgical treatment, which only increases morbidity associated with the combination of surgery and chemoradiotherapy [4]. This is reflected in our results, as the systematic use of pelvic MRI was associated with a reduction in the upstaging rate from 31.6% before 2010 to 4.4% in the subsequent period. This improvement may partly explain the higher PFS observed in patients operated on from 2010 onward. Although PFS improved after 2010, this did not translate into an OS benefit. One plausible explanation is lead-time bias, due to more sensitive imaging detecting progression earlier without affecting OS. This should be taken into account when interpreting these findings and should be approached with caution. These results also support the role of high-quality preoperative assessment and multidisciplinary planning in optimizing treatment strategies. Similar trends have been reported in other malignancies, where adherence to guideline-based surgical quality indicators correlates with reduced morbidity and improved oncologic results [20].

Despite these improvements, we identified areas requiring further standardization. Notably, QI 7, which mandates reporting of parametrial tissue measurements in two dimensions, and QI 8, requiring structured prospective follow-up and morbidity reporting, were not consistently met in our cohort, emphasizing the need for improved documentation processes in our clinical practice and future studies. In contrast the Chinese cohort met follow-up standards, achieving a ≥90% structured prospective reporting of complications and follow-up whereas our finding of insufficient compliance indicates a clear area for process improvement [8]. Similarly, improvements are needed in the documentation of the surgical procedure at our center (QI 6). Conversely, the Chinese study achieved 100% completeness for both surgical and pathology reports, highlighting institutional variability in documentation rigor [8]. Addressing these gaps may facilitate more robust quality assurance and enable benchmarking against other centers.

Regarding QIs related to the quality of surgical procedures, in our cohort we also observed that the overall proportion of patients with positive margins and parametrial invasion (QI 10) exceeded the threshold established by the ESGO QIs. However, when focusing on the post-2010 group analysis, this indicator was met, as only 1.1% of patients had positive margins and none showed parametrial involvement.

### 4.1. Results in the Context of the Published Literature

The literature evaluating the relevance of ESGO QIs in the surgical management of cervical cancer is limited. The most comprehensive study to date was conducted by Ding et al. [8], who performed a retrospective cohort analysis of more than 7000 cases treated between 2014 and 2019 at a high-volume hospital in China. Their findings demonstrated overall adherence to the majority of ESGO QIs, underscoring the feasibility of applying these standards in large clinical settings. However, two key indicators emphasized in our study—multidisciplinary case discussion and the systematic use of preoperative pelvic MRI—were not achieved in their cohort, as 80.3% of patients did not undergo MRI and none of the cases were discussed in a multidisciplinary team. These findings suggest that strict adherence to ESGO quality indicators, particularly structured preoperative evaluation, and multidisciplinary planning, can directly influence surgical precision and oncologic outcomes, highlighting the critical role of quality assurance in cervical cancer management.

Boria et al. performed another retrospective cohort study across multiple European centers, analyzing the characteristics, outcomes, and compliance with ESGO QIs in patients undergoing radical hysterectomy for stage IB1 (FIGO 2009) cervical cancer [7]. Their results showed high adherence to most QIs, particularly in high-volume centers, and highlighted that compliance with guideline-based surgical standards was associated with improved perioperative outcomes and PFS, which is consistent with the findings from our cohort.

In our multivariate analysis, cervical conization prior to radical hysterectomy was observed to act as a protective factor against recurrence in patients with cervical cancer. These findings are consistent with previous reports and may be explained by the reduction in tumor volume, which could prevent tumor dissemination during surgery. Benoit et al. conducted one of the first studies specifically analyzing the effect of cervical conization in early-stage cervical cancer [21]. While univariate analysis suggested an association between conization and reduced recurrence risk, statistical significance was not maintained in the multivariate model. In contrast, two retrospective studies by Casarin et al. found, similarly to our results, that cervical conization was protective against recurrence (HR 0.29, 95% CI 0.13–0.91; *p* = 0.03) [22,23]. Their multivariate analysis also demonstrated that the presence of residual tumor in the conization specimen was associated with a five- to six-fold increased risk of recurrence. Likewise, in the study by Bizarri et al., which applied a propensity score matching method to balance groups, patients who underwent conization exhibited significantly higher PFS compared to those who did not (89.8% vs. 80%, *p* = 0.010) [24]. Finally, two studies from the SUCCOR group (SUCCOR Cone and SUCCOR Risk) also identified cervical conization as a protective factor against recurrence in patients with stage IB1 cervical cancer undergoing radical hysterectomy [25,26].

### 4.2. Limitations and Strengths of the Study

Several limitations should be acknowledged. First, the retrospective nature of the study and the inclusion of patients from a single center may introduce potential, unmeasured bias. Second, the limited number of patients operated on per year, and the smaller case number in the pre-2010 group, may have influenced the results obtained. In addition, changes in surgical practice after the publication of the LACC trial in 2018, including the abandonment of uterine manipulators and the restriction of minimally invasive surgery to small tumors, may also have influenced the outcomes of patients treated in the later period. However, our detailed evaluation of QIs adherence over nearly two decades offers valuable real-world insights into the progressive implementation of ESGO guidelines and their association with patient outcomes. The large number of univariate comparisons increases the risk of chance findings; we mitigated this by prioritizing the Cox multivariable model and reporting effect sizes with confidence intervals.

It should be noted that the confidence intervals for some variables in the multivariate model are extremely wide, reflecting a high degree of uncertainty. This likely results from the limited number of events for certain covariates. Therefore, these findings should be interpreted with caution.

Future prospective multicenter studies are warranted to validate these observations, refine quality indicators, and evaluate their impact on long-term survival and quality of life.

Surgical procedures performed exclusively by ESGO-certified gynecologic oncologists represent a major strength of our practice, ensuring that all patients were managed by highly specialized surgeons, in line with international recommendations. Other significant strength of this analysis lies in the features of the data presented. We systematically reported absolute numbers and percentages for each indicator, clearly identifying whether ESGO benchmarks were met. Furthermore, this evaluation extends beyond outcome measures, incorporating detailed assessments of structural and procedural aspects, which are often underreported in quality audits. Such an approach provides a more holistic understanding of institutional performance and highlights areas for targeted improvement.

## 5. Conclusions

In summary, our results suggest that the introduction of ESGO QIs, particularly standardized pelvic MRI for staging and multidisciplinary tumor board evaluation, was associated with improved surgical and oncological outcomes in cervical cancer. In addition, cervical conization prior to radical hysterectomy emerged as a protective factor for recurrence. The correct application of ESGO QIs, along with appropriate staging and pathological assessment, is essential to improve prognosis in cervical cancer.

## Figures and Tables

**Table 1:** Compliance with ESGO QIs in Cervical Cancer Surgery at Our Center.

No	ESGO QI	Target Required	Value in Our Cohort	Achieved
**1**	Number of radical procedures in cervical cancer performed per center per year	≥15	7.1 (mean)	No
**2**	Surgery performed or supervised by a certified gynecologic oncologist or trained surgeon dedicated to gynecologic cancer	100%	100%	Yes
**3**	Center participating in ongoing clinical trials in gynecologic cancer	≥1	Data not available before 2010 100% since 2010	Yes (from 2010)
**4**	Treatment discussed at a multidisciplinary team meeting	100%	0% before 2010 100% since 2010	Yes (from 2010)
**5**	Required pre-operative investigation (pelvic MRI)	100%	79% overall 100% since 2010	No Yes (from 2010)
**6**	Minimum required elements in surgical reports	100%	57%	No
**7**	Minimum required elements in pathology and pathology reports	≥90%	0% (1 parameter never reported)	No
**8**	Structured prospective reporting of follow-up and 30-day post-operative morbidity	≥90%	0%	No
**9**	Urological fistula rate within 30 days after radical parametrectomy	≤3%	2.38%	Yes
**10**	Proportion of patients with clear vaginal and parametrial margins	≥97%	95.3% overall 98.9% after 2010	No overall Yes (from 2010)
**11**	Proportion of patients with stage T1b disease upstaged after surgery	<10%	12.5% overall 4.4% since 2010	No overall Yes (from 2010)
**12**	Recurrence rate at 2 years in stage pT1b1N0 patients	<10%	9.97%	Yes
**13**	Proportion of patients with stage T1 disease undergoing lymph node staging	≥98%	100%	Yes
**14**	Counseling about the possibility of fertility-sparing treatment (FST)	100%	100%	Yes
**15**	Proportion of patients receiving adjuvant chemoradiotherapy after pT1b1pN0 disease	<15%	3.13%	Yes

ESGO: European Society of Gynaecological Oncology; QI: quality indicator.

**Table 2:** Baseline, surgical and pathological characteristics from patients (n = 128) stratified by year of surgery (before vs. after 2010). Data are given in median [interquartile range] or frequencies (relative percentages).

	Total (n = 128)	Before 2010 (n = 38)	2010 or After (n = 90)	*p*-Value
**Age (years)**	128	46.0 ± 9.8	48.1 ± 12.3	0.3684
**BMI (kg/m^2^)**	121	25.9 ± 7.2	26.5 ± 4.7	0.5751
**FIGO Stage (2018)**				
IA1	3 (2.3%)	2 (5.3%)	1 (1.1%)	0.057
IA2	6 (4.7%)	3 (7.9%)	3 (3.3%)	
IB1	46 (35.9%)	9 (23.7%)	37 (41.1%)	
IIA1	5 (3.9%)	0 (0.0%)	5 (5.6%)	
IB2	68 (53.1%)	24 (63.2%)	44 (48.9%)	
**Maximum size per image (mm)**	90	24 ± 17.7	14.4 ± 12.7	0.0495
**Previous Conization**				
Yes	66 (51.6%)	24 (63.2%)	38 (42.2%)	0.035
No	62 (48.4%)	14 (36.8%)	52 (48%)	
**Type of procedure**				
Radical Hysterectomy	114 (89.1%)	36 (4.7%)	78 (86.7%)	0.183
Radical Trachelectomy	12 (9.4%)	1 (2.6%)	11 (12.2%)	
Radical Parametrectomy	2 (1.6%)	1 (2.6%)	1 (2.6%)	
**Type of parametrial resection**				
Type A	2 (1.5%)	2 (5.3%)	0 (0%)	<0.001
Type B	43 (33.6%)	1 (2.6%)	42 (46.7%)	
Type C1	81 (63.3%)	35 (92.1%)	46 (51.1%)	
No registered	2 (1.5%)	0(0%)	2 (2.2%)	
**Surgical approach**				
Open	21 (16.4%)	8 (21.1%)	13 (14.4%)	0.603
Laparoscopic	106 (82.8%)	30 (79.0%)	76 (84.4%)	
Robotic	1 (0.8%)	0 (0%)	1 (1.1%)	
**Intraoperative complications**				
Yes	11 (8.6%)	6 (15.8%)	5 (5.6%)	0.047
**Early postoperative complications (<30 days)**				
Yes	42 (32.8%)	11 (29.0%)	31 (34.4%)	0.575
**Histological Subtype**				
Squamous	81 (63.3%)	23 (60.5%)	58 (64.4%)	0.605
Adenocarcinoma	25 (19.5%)	7 (18.4%)	18 (20.1%)	
Other	22 (17.2%)	8 (21.1%)	14 (15.5%)	
**Grade**				
Grade 1	32 (25.0%)	11 (29.0%)	21 (23.3%9	0.660
Grade 2	64 (50.0%)	16 (42.1%)	48 (53.3%)	
Grade 3	30 (23.4%)	10 (26.3%)	20 (22.2%)	
No registered	2 (1.6%)	1 (2.6%)	1 (1.1%)	
**LVSI**				
Yes	35 (27.3%)	17 (44.7%)	18 (20.0%)	0.005
No	91 (71.2%)	20 (52.7%)	71 (78.9%)	
No registered	2 (1.5%)	1 (2.6%)	1 (1.1%)	

BMI: body mass index. LVSI: lymph vascular space invasion.

**Table 3:** Cox multivariate analysis for PFS.

	PFS		
	HR	CI 95%	*p* Value
**Age (years)**	0.97	0.92–1.03	0.300
**BMI (kg/m^2^)**	0.96	0.84–1.09	0.525
**Maximum tumor diameter**	1.03	0.93–1.14	0.597
**Tumoral Grade (Ref. 1)**			
2	0.40	0.06–2.52	0.328
3	1.52	0.27–8.72	0.635
**FIGO 2018 histological stage (Ref. IA2)**			
IA1	-	-	1.000
IB1	-	-	1.000
IB2	0.05	0.001–2.63	0.140
IB3	0.03	<0.001–16.43	0.275
IIB	0.002	<0.001–0.38	0.200
IIIC1	0.04	<0.001–4.15	0.177
**LVSI**	3.38	0.92–12.38	0.066
**Stromal invasion (Ref. superficial)**			
Middle 1/3	1.47	0.11–19.85	0.771
Deep 1/3	2.09	0.20–22.30	0.541
**Positive margins**	5.81	0.14–234.10	0.351
**Parametrial invasion**	9.00	0.23–358.98	0.243
**Previous conization**	**0.12**	0.02–0.73	**0.021**
**Positive lymph nodes**	0.48	-	-
**Diagnostic group (Ref. <2010)**	1.13	0.30–4.28	0.859

PFS: progression-free survival; HR: hazard ratio; CI: confidence interval; BMI: body mass index; FIGO: International Federation of Gynecology and Obstetrics; LVSI: lymph vascular space invasion.

## Data Availability

The data presented in this study are available on request from the corresponding authors. The availability of the data is restricted to investigators based in academic institutions.

## Abbreviations

The following abbreviations are used in this manuscript:

ESGO	European Society of Gynaecological Oncology
QI	Quality Indicators
PFS	Progression Free Survival
HPV	Human papillomavirus
PL	Pelvic lymphadenectomy
SLNB	Sentinel lymph node biopsy
MRI	Magnetic resonance imaging
SD	Standard deviation
IQR	Interquartile range
EBR	External beam radiation
BT	Vaginal brachytherapy
CRT	Chemoradiotherapy
MDT	Multidisciplinary team
